# vWF/ADAMTS13 is associated with on-aspirin residual platelet reactivity and clinical outcome in patients with stable coronary artery disease

**DOI:** 10.1186/s12959-017-0151-3

**Published:** 2017-11-22

**Authors:** Ellen M. K. Warlo, Alf-Åge R. Pettersen, Harald Arnesen, Ingebjørg Seljeflot

**Affiliations:** 1 Center for Clinical Heart Research, Department of Cardiology, Oslo University Hospital, Ullevaal, Pb 4956 Nydalen, 0424 Oslo, Norway; 2 Faculty of Medicine, University of Oslo, Oslo, Norway; 3 Center for Heart Failure Research, University of Oslo, Oslo, Norway; 4 Department of Medicine, Vestre Viken HF, Ringerike Hospital, Hønefoss, Norway

**Keywords:** ADAMTS13, Von Willebrand factor, Aspirin, Residual platelet reactivity, Cardiovascular disease

## Abstract

**Background:**

The mechanisms behind residual platelet reactivity (RPR) despite aspirin treatment are not established. It has been shown that coronary artery disease (CAD) patients with high on-aspirin RPR have elevated levels of von Willebrand factor (vWF). ADAMTS13 is a metalloprotease cleaving ultra large vWF multimers into less active fragments.

Our aim was to investigate whether ADAMTS13 and vWF/ADAMTS13 ratio were associated with high RPR, and further with clinical endpoints after 2 years.

**Methods:**

Stable aspirin-treated CAD patients (*n* = 999) from the ASCET trial. RPR was assessed by PFA-100. ADAMTS13 antigen and activity were analysed using chromogenic assays. Endpoints were a composite of acute myocardial infarction, stroke and death.

**Results:**

The number of patients with high RPR was 258 (25.8%). Their serum thromboxane B_2_ (TxB_2_) levels were low, indicating inhibition of COX-1. They had significantly lower levels of ADAMTS13 antigen compared to patients with low RPR (517 vs 544 ng/mL, *p* = 0.001) and significantly lower ADAMTS13 activity (0.99 vs 1.04 IU/mL, *p* = 0.020). The differences were more pronounced when relating RPR to ratios of vWF/ADAMTS13 antigen and vWF/ADAMTS13 activity (*p* < 0.001, both). We found an inverse correlation between vWF and ADAMTS13 antigen (*r* = −0.14, p < 0.001) and ADAMTS13 activity (*r* = −0.11, p < 0.001). No correlations between TxB_2_ and ADAMTS13 antigen or activity, were observed, implying that ADAMTS13 is not involved in TxB2 production. Patients who experienced endpoints (*n* = 73) had higher vWF level (113 vs 105%, *p* = 0.032) and vWF/ADAMTS13 antigen ratio (0.23 vs 0.20, *p* = 0.012) compared to patients without. When dichotomizing vWF/ADAMTS13 antigen at median level we observed that patients above median had higher risk for suffering endpoints, with an adjusted OR of 1.86 (95% CI 1.45, 2.82).

**Conclusion:**

These results indicate that ADAMTS13 is of importance for RPR, and that it in combination with vWF also is associated with clinical endpoints in stable CAD patients on aspirin.

**Trial registration:**

Clinicaltrials.gov NCT00222261. Registered 13.09.2005. Retrospectively registered.

**Electronic supplementary material:**

The online version of this article (10.1186/s12959-017-0151-3) contains supplementary material, which is available to authorized users.

## Background

Von Willebrand factor (vWF) is an established marker of endothelial activation, and patients with elevated plasma levels of the ultra large vWF molecules are at high risk for future cardiovascular events [1–4].

vWF is a glycoprotein involved in both platelet activation and aggregation through its binding sites for GpIb and GpIIb/IIIa, respectively [5]. It is released to the circulation as ultra-large multimers from Weibel-Palade bodies in the endothelial cells, as well as from α-granules in platelets. vWF occurs in different lengths in the circulation and its thrombotic properties differ depending on its size [6]. It is involved in haemostasis when released as ultra-large multimers, and the activity decreases as the size abates [5]. vWF’s activity is also enhanced by shear stress, promoting conformational changes of the molecule to expose important binding sites [6]. vWF is cleaved into smaller fragments with reduced thrombotic activity by a metalloproteinase, ADAMTS13, “A Disintegrin And Metalloprotease with TromboSpondin type 1 motif, member 13” [7]. Reduced amount and activity of ADAMTS13 lead to less fragmentation of vWF with subsequent higher amount of the ultra-large vWF molecules, thus potentially a more prothrombotic state.

The role of ADAMTS13 in coronary artery disease (CAD) has not been established, and inconclusive results have been reported [3]. A recent meta-analysis showed low ADAMTS13 levels, both amount and activity, with concomitant ultra-large vWF, to be a risk factor for myocardial infarction [8]. The importance of ADAMTS13 for platelet reactivity, indirectly by acting on vWF or directly, is limited described.

Aspirin is a cornerstone in treatment of cardiovascular disease (CVD), but patients on aspirin may still experience new cardiovascular events. The phenomenon of residual platelet reactivity (RPR) despite use of aspirin has been extensively studied with diverging results [9]. The prevalence of high RPR and any predictive value vary considerably depending on the laboratory methods used [10, 11]. Thus, there is no recommendation to introduce platelet function testing in clinical practice. Regardless of this, the mechanisms behind high RPR assessed by platelet function testing are still not known in details. In the Aspirin Nonresponsiveness and Clopidogrel Endpoint Trial (ASCET) [12], it was shown that patients with high on-aspirin RPR had significantly elevated levels of vWF compared to patients with low RPR [13].

The aim of the present study was to investigate whether ADAMTS13 alone and as a vWF/ADAMTS13 ratio were associated with the presence of high RPR in aspirin treated CAD patients. We hypothesised that low ADAMTS13 would contribute to high RPR. We further explored whether ADAMTS13 and the vWF/ADAMTS13 ratio were associated with disease entities and risk factors and further with clinical outcome after 2 years follow-up.

## Methods

### Study population

This is a substudy of the ASCET trial which was performed at Center for Clinical Heart Research, Oslo University Hospital, Ullevaal, Oslo, Norway, from March 2003 until July 2010 [12]. The design of the trial has previously been published [14], and it is registered at https://www.clinicaltrials.gov/ (identification No. NCT00222261).

Patients (*n* = 1001) with stable CAD, previously verified by angiography, were included. All patients were on single antiplatelet therapy with aspirin 160 mg/d for at least 1 week prior to inclusion. Patients were randomized to either continue on aspirin 160 mg/d or change to clopidogrel 75 mg/d and were followed for 2 years for clinical endpoints. Patients in need of dual antiplatelet therapy or warfarin were excluded. The ASCET study was approved by the regional ethics committee and all patients gave their written consent.

Current smokers were defined as patients still smoking or former smokers who had quit less than 3 months ago. Hypertensives included patients with treated hypertension. The criteria for diabetes were patients with treated T1DM and T2DM or fasting blood glucose > 7 mmol/L.

Clinical endpoints in the main ASCET study included unstable angina pectoris, myocardial infarction (MI), non-haemorrhagic stroke and death. For the purpose of the present investigation we excluded the group of unstable angina pectoris as this is a less defined diagnosis. Thus, the recorded endpoints were MI, non-haemorrhagic stroke and death. Endpoints were recorded at study visits and on request for patients unable to attend the final visit. An endpoint committee performed the evaluation of endpoints and internationally accepted diagnostic criteria were used.

### Blood sampling

Blood samples were drawn at inclusion in fasting condition between 08.00 and 10.30 AM, 24 h after last intake of aspirin. Citrated plasma (0.129 M in dilution 1:10) was stored on ice and separated within 30 min by centrifugation at 4 °C and 3000 g for 20 min, and stored at -80 °C until analysed for ADAMTS13 antigen and activity, performed by commercial methods (IMUBIND® (Seksui Diagnostics GmbH, Pfungstadt, Germany and TECHNOZYM® ADAMTS-13 activity assay, Technoclone, Vienna, Austria, respectively). We have previously reported on vWF, determined in citrated plasma by use of Asserachrom vWF Ag (Stago Diagnostica, Asnieres, France), and Thromboxane B_2,_ analysed in serum prepared from whole blood without anticoagulants and kept at 37° for 1 h before centrifugation at 2500×g for 10 min (Amersham Thromboxane B_2_ Biotrak Assay, GE Healthcare, Buckinghamshire, UK) [12, 13].

### Residual platelet reactivity (RPR)

The method has been described in details previously [12]. Briefly, citrated whole blood (0.129 M in dilution 1:10) was analysed within 2 h by the PFA100 system (Siemens Healthcare Diagnostics, Germany). This system stimulates platelet-based haemostasis in vitro by use of cartridges with collagen and epinephrine. Closure time (CT) was recorded to determine platelet function. The cut-off value, determined by testing 200 CAD patients not on antiplatelet therapy, was set at 196 s based on the 95 percentile in this cohort [15]. Patients with CT below this level were classified with high RPR and patients above this level with low RPR.

### Statistical analysis

Continuous variables are presented as mean ± SD or median (25th, 75th percentiles) when appropriate. Categorical data are presented as numbers or percentages. Students unpaired t-test or Mann-Whitney U test were used to compare continuous variables between groups, and group comparisons for categorical variables were performed by Pearson’s chi-squared test. Correlation analyses were performed by Spearman’s rho. Logistic regression analysis was used to adjust for relevant covariates, estimated from Additional file 1 Table S1. A *p*-value < 5% was considered statistically significant. SPSS version 22 (SPSS Inc., IL, USA) was used.

## Results

Baseline characteristics of the total population and according to the presence of low or high RPR are shown in Table 1. Blood samples from two patients were not available for ADAMTS13 analyses, thus all results are given for 999 patients. The number of patients with high RPR was 258 (25.8%). TxB_2_ levels were low in all patients, compared to levels in individuals not on aspirin [15]. There was a significantly lower percentage of hypertensives, and a higher proportion of smokers in the high RPR group compared to the group with low RPR. There was no significant difference in clinical endpoints with regard to high and low RPR. These data on patients with high and low RPR have previously been published [12, 13].

**Table 1:** Baseline characteristics of the total populating and according to the presence of low or high RPR

	Total population	Low RPR (*n* = 741)	High RPR (*n* = 258)	*p*-value^§^
Age (years)^a^	62.4 (36–81)	62.6 (36–81)	61.7 (41–80)	0.154
Sex, female, n (%)	218 (21.8)	159 (21.5)	58 (22.5)	0.731
Caucasian, n (%)	968 (96.8)	724 (97.7)	243 (94.2)	**0.006**
Cardiovascular risk factors, n (%)				
Current smoking	203 (20.3)	136 (18.4)	67 (26.0)	**0.009**
Hypertension	556 (55.7)	433 (58.4)	123 (47.9)	**0.003**
Diabetes mellitus	200 (20.0)	148 (20.0)	52 (20.2)	0.950
Previous CVD^d^	635 (63.6)	462 (62.3)	172 (66.9)	0.189
- Previous myocardial infarction	436 (43.7)	314 (42.4)	121 (47.1)	0.195
Systolic blood pressure (mmHg)^b^	140 ± 19	141 ± 19	136 ± 19	**< 0.001**
Diastolic blood pressure (mmHg)^b^	82 ± 10	82 ± 10	81 ± 9	**0.040**
Body mass index (kg/m^2^)^b^	27.4 ± 3.7	27.5 ± 3.8	27.2 ± 3.5	0.237
Blood tests				
Total cholesterol (mmol/L)^b^	4.55 ± 0.98	4.55 ± 0.99	4.53 ± 0.96	0.793
LDL cholesterol (mmol/L)^b^	2.53 ± 0.83	2.53 ± 0.83	2.53 ± 0.83	0.998
HDL cholesterol (mmol/L)^b^	1.33 ± 0.41	1.34 ± 0.42	1.31 ± 0.37	0.277
Triglycerides (mmol/L)^c^	1.31 (0.93, 1.84)	1.29 (0.91, 1.84)	1.35 (0.97, 1.85)	0.144
Residual platelet reactivity, n (%)	258 (25.8)			
TxB_2_ (ng/mL)^a^	2.7 (0–21)	2.6 (0–21)	3.0 (0–15)	0.102
Medication, n (%)				
Statins	982 (98.3)	727 (98.2)	254 (98.4)	0.825
B-blockers	755 (75.8)	561 (76.1)	193 (74.8)	0.672
Calcium channel blockers	255 (25.6)	196 (26.5)	59 (23.0)	0.272
ACE-inhibitors	263 (26.5)	200 (27.1)	63 (24.7)	0.455
ARB	239 (24.0)	187 (25.3)	52 (20.4)	0.111

*CVD* cardiovascular disease, *MI* myocardial infarction, *PCI* percutaneous coronary intervention, *CABG* coronary artery bypass graft, *TxB*_*2*_ thromboxane B_2_, *ACE* angiotensin-converting enzyme, *ARB* angiotensin II receptor blockers

^**§**^ *p*-values refer to differences between the groups with high or low RPR

^a^mean (range) ^b^mean ± SD ^c^median (25th, 75th percentiles) ^d^Previous MI, PCI, CABG, non-haemorrhagic stroke

Significant p-values are highlighted with boldface

### ADAMTS13 as related to RPR

Levels of ADAMTS13 antigen and activity are shown in Table 2, in the total cohort and according to the RPR status. Patients with high RPR had significantly higher levels of vWF (*p* < 0.001) and platelet count (*p* = 0.010) compared to those with low RPR, as previously published [13]. In the present investigation we observed that patients with high RPR had significantly lower levels of both ADAMTS13 antigen and activity compared to individuals with low RPR (*p* = 0.001, *p* = 0.020, respectively). When calculating ratios of vWF/ADAMTS13 antigen and vWF/ADAMTS13 activity the differences were even more pronounced, showing both ratios to be significantly higher in patients with high RPR (*p* < 0.001 for both).

**Table 2:** Baseline levels of the measured markers in the total population and according to the presence of low or high RPR

	Total population	Low RPR (*n* = 741)	High RPR (*n* = 258)	*p*-value^§^
vWF (%)	105 (82, 133)	100 (79, 126)	124 (94, 145)	**< 0.001**
ADAMTS13 antigen (ng/mL)	532 (461, 606)	537 (469, 613)	511 (448, 580)	**0.001**
ADAMTS13 activity (IU/mL)	1.03 (0.83, 1.19)	1.04 (0.84, 1.19)	0.99 (0.76, 1.16)	**0.020**
Ratio vWF/ADAMTS13 antigen	0.20 (0.15, 0.26)	0.19 (0.14, 0.25)	0.23 (0.18, 0.31)	**< 0.001**
Ratio vWF/ADAMTS13 activity	107 (77, 152)	101 (73, 137)	127 (93, 192)	**< 0.001**
Platelet count (× 10^9^/L)	227 (195, 264)	224 (192, 261)	236 (200, 273)	**0.010**

Values are given as median (25th, 75th percentiles)

^**§**^ *p*-values refer to differences between the groups with high or low RPR

Significant p-values are highlighted with boldface

Statistically significant, but weak inverse correlations were found between the levels of vWF and ADAMTS13 antigen (*r* = −0.14, *p* < 0.001) and ADAMTS13 activity (*r* = −0.11, p < 0.001). ADAMTS13 antigen and activity were significantly inter-correlated (*r* = 0.47, p < 0.001).

### ADAMTS13 as related to disease entities

Table 3 shows ADAMTS13 antigen and activity as related to different cardiovascular risk factors. When dichotomizing age at median level, we found that patients above median (62 years) had higher levels of vWF, lower ADAMTS13 antigen and activity, and higher ratios (*p* < 0.001, for all). No sex differences were observed in the measured markers. Diabetic patients had significantly higher levels of both vWF (*p* = 0.023) and ADAMTS13 antigen (*p* = 0.003), whereas hypertensive patients had significantly lower levels of vWF (*p* = 0.033) and lower ADAMTS13 activity (*p* = 0.034) compared to normotensives. Patients with previous CVD had significantly higher levels of vWF (p < 0.001) and also higher vWF/ADAMTS13, both antigen ratio (*p* = 0.001) and activity ratio (*p* = 0.002). We observed no differences between smokers and non-smokers.

**Table 3:** Baseline levels of the measured markers according to different clinical conditions

		n	vWF (%)	ADAMTS13 antigen (ng/mL)	ADAMTS13 activity (IU/mL)	Ratio antigen	Ratio activity
Age > median (62 years)	+	500	110 (88, 137)	519 (455, 581)	1.00 (0.79, 1.15)	0.21 (0.17, 0.29)	117 (86, 170)
	–	500	100 (79, 127)	547 (467, 627)	1.05 (0.90, 1.21)	0.19 (0.14, 0.25)	97 (70, 136)
	p		**< 0.001**	**< 0.001**	**< 0.001**	**< 0.001**	**< 0.001**
Sex (female)	+	218	106 (82, 136)	549 (463, 622)	1.04 (0.84, 1.19)	0.20 (0.14, 0.26)	111 (76, 157)
	–	782	106 (83, 133)	525 (460, 596)	1.02 (0.82, 1.19)	0.20 (0.15, 0.27)	106 (77, 150)
	p		0.840	0.052	0.877	0.611	0.726
Diabetes	+	200	111 (83, 141)	557 (465, 639)	1.05 (0.81, 1.18)	0.20 (0.15, 0.27)	117 (84, 170)
	–	800	105 (83, 131)	524 (459, 596)	1.02 (0.83, 1.19)	0.20 (0.15, 0.26)	106 (76, 147)
	p		**0.023**	**0.003**	0.931	0.602	0.059
Hypertension	+	556	104 (80, 132)	530 (463, 606)	1.01 (0.79, 1.16)	0.20 (0.15, 0.26)	107 (76, 150)
	–	443	108 (86, 134)	533 (458, 607)	1.05 (0.86, 1.21)	0.20 (0.16, 0.28)	106 (77, 153)
	p		**0.033**	0.959	**0.034**	0.081	0.962
Previous CVD	+	635	110 (86, 137)	534 (463, 608)	1.02 (0.82, 1.19)	0.20 (0.16, 0.27)	110 (82, 160)
	–	364	98 (78, 126)	525 (456, 602)	1.04 (0.85, 1.18)	0.19 (0.14, 0.25)	103 (72, 136)
	p		**< 0.001**	0.453	0.663	**0.001**	**0.002**
Smokers	+	203	107 (88, 134)	520 (441, 590)	1.03 (0.86, 1.19)	0.20 (0.16, 0.28)	108 (78, 151)
	–	796	105 (83, 133)	532 (466, 607)	1.02 (0.82, 1.18)	0.20 (0.15, 0.26)	107 (77, 152)
	p		0.621	0.100	0.306	0.515	0.796

Values are given as median (25th, 75th percentiles)

*p*-values refer to differences in the measured marker between patients having the specified clinical condition or not

Significant p-values are highlighted with boldface

### ADAMTS13 as related to clinical endpoints

After 2 years follow-up 73 clinical endpoints (MI, stroke or death) were recorded. These patients had higher levels of vWF and vWF/ADAMTS13 antigen ratio at inclusion compared to patients without endpoints (Table 4). We found no significant differences in the levels of ADAMTS13, neither antigen nor activity.

**Table 4:** Baseline levels of the measured markers according to clinical endpoint or no endpoint

	Endpoint (*n* = 73)	No endpoint (*n* = 927)	*P*-value^§^
vWF, %	113 (93, 141)	105 (81, 133)	**0.032**
ADAMTS-13 antigen, ng/mL	506 (452, 576)	533 (462, 607)	0.147
ADAMTS-13 activity, IU/mL	1.00 (0.73, 1.13)	1.03 (0.83, 1.19)	0.212
Ratio antigen	0.23 (0.17, 0.29)	0.20 (0.15, 0.26)	**0.012**
Ratio activity	119 (88, 175)	107 (77, 150)	0.051
Platelet count (× 10^9^/L)	221 (181, 272)	228 (196, 263)	0.446

Values are given as median (25th, 75th percentiles)

^**§**^ *p*-values refer to differences between the groups with high or low RPR

Significant p-values are highlighted with boldface

**Fig. 1:**
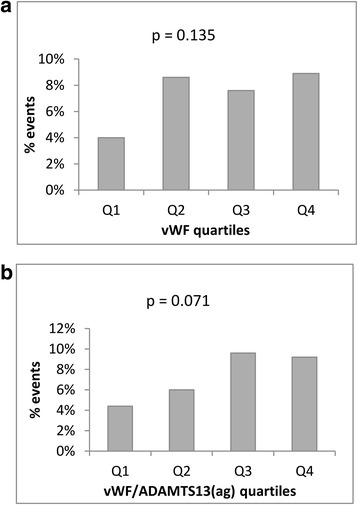
**a** Endpoints during 2 years follow-up according to quartiles of vWF. *p*-value refers to trend analysis. **b** Endpoints during 2 years follow-up according to quartiles of vWF/ADAMTS13 antigen ratio. *p*-value refers to trend analysis

When dividing vWF and vWF/ADAMTS13 antigen ratio levels into quartiles, there were no significant trends for frequency of endpoints (*p* = 0.135, *p* = 0.071, respectively) (Fig. 1). Nevertheless, we observed a potential cut-off level at the lowest quartile for vWF (Fig 1a). When dichotomizing vWF at this level, a higher event rate was demonstrated in patients with levels in the three upper quartiles compared to those in the lowest quartile with an OR of 2.18 (95% CI 1.10, 4.31). The significance was, however, lost after adjusting for the covariates age, sex, diabetes, and previous CVD (*p* = 0.077).

When dichotomizing vWF/ADAMTS13 antigen at the median value (Fig 1b), an OR of 1.89 (95% CI 1.15, 3.11) for suffering an event when having levels above the median was observed (*p* = 0.011). After adjustments for covariates the OR was 1.68 (95% CI 1.01, 2.80) and the significance was retained (*p* = 0.045). Additional adjustment for the randomized treatment principle did not influence the results.

## Discussion

This study was performed in a stable CAD population where 258 (25.8%) patients had high RPR, determined by the PFA-100 method. TxB_2_ levels were low, indicating adequate aspirin compliance. High RPR patients had higher levels of vWF compared to patients with low RPR as previously published [13].

Our main findings were that patients with high RPR had significantly lower levels of ADAMTS13, both antigen and activity, and higher vWF/ADAMTS13 ratios, and further significantly higher vWF levels and vWF/ADAMTS13 antigen ratio in patients who experienced a clinical endpoint after 2 years compared to patients who did not.

To our knowledge, this is the first study reporting on the importance of ADAMTS13 on RPR in stable aspirin-treated CAD patients. Our results are mostly in line with a similar study by Marcucci et al. using other methods for RPR in a smaller CAD population, however, performed in the acute phase [16]. They observed significantly higher vWF and lower ADAMTS13 activity, but no difference in ADAMTS13 antigen in patients with high RPR. The different results on ADAMTS13 antigen might be due to different population sizes, but also the acute phase with a high degree of inflammation, which is known to affect both vWF and ADAMTS13 levels [17].

In our study RPR was related to both the amount and the activity of ADAMTS13. A possible mechanism is that low ADAMTS13 levels cause less fragmentation of vWF, resulting in more long vWF molecules that can activate platelets, thereby a higher RPR. We do not know whether high vWF levels per se cause high RPR or only act as a covariate. In the present situation the association between vWF and ADAMTS13 may be due to the reciprocal course of these molecules, i.e. high vWF may lead to low ADAMTS13 as a result of the increased availability of vWF which may lead to an “exhaustion” or elimination of ADAMTS13 by unknown mechanisms [17].

We observed only a weak correlation between vWF and ADAMTS13 which is in accordance with other reports, showing either weak or no correlation [18–20]. Even though it is well established that ADAMTS13 cleaves vWF, both are affected by other factors that might weaken this correlation [21–23]. It has also been suggested that the two molecules, independent of each other, are associated with clinical endpoints, and that ADAMTS13 have cardioprotective properties beyond vWF cleavage [20, 24, 25].

Both vWF and ADAMTS13, as well as the ratios were significantly associated with age, in line with other reports [26–30]. No significant sex differences were observed. Lower ADAMTS13, both antigen and activity have previously been shown in men [27–30]. Higher levels of both vWF and ADAMTS13 antigen were observed in diabetic patients. High vWF have generally been associated with diabetes, but the results for ADAMTS13 are more diverging [20, 29–31]. In the Rotterdam study higher ADAMTS13 activity in patients with diabetes compared to those without was reported [29], and they also showed ADAMTS13 activity to be an independent risk factor for both prediabetes and diabetes type 2 [32]. In hypertensive patients higher vWF and lower ADAMTS13 activity were found, which to some degree is in accordance with other reports, although varying results exist [26, 31]. Also, patients with previous CVD had higher vWF levels in our study. There are some case-control studies supporting this result, whereas the observations on ADAMTS13 are diverging [19, 20]. We found no differences between smokers and non-smokers which is in accordance with previous reports [20, 31].

Our observations of vWF and ADAMTS13 to be associated with clinical endpoints are partly in accordance with previous reports. vWF have repeatedly been associated with increased risk of coronary heart disease [3]. We observed higher levels of vWF in patients experiencing clinical endpoints, however, the significance was lost when adjusting for covariates. This might strengthen the recent suggestion that vWF is more modestly associated with coronary heart disease than previously estimated [32].

Previous reports on ADAMTS13, both antigen and activity as related to clinical outcome are inconclusive, and we could not demonstrate any association between ADAMTS13 and clinical endpoints. The meta-analysis by Maino et al. concluded that ADAMTS13 levels below the 5th percentile was associated with increased risk of MI [8], whereas Sonneveld et al. concluded an uncertainty on whether ADAMTS13 increases the risk of arterial thrombosis due to diverging results and lack of prospective studies [3].

We could, however, demonstrate that patients who suffered a clinical endpoint had significantly higher levels of vWF/ADAMTS13 antigen ratio. Patients with ratio above median level had an OR of 1.68 for suffering an event, significant also after adjustments for relevant covariates, indicating that the combination of vWF and ADAMTS13 antigen might be a better prognostic marker than vWF alone. To the best of our knowledge this is the first report on vWF/ADAMTS13 antigen ratio in relation to future cardiovascular events.

### Study limitations

The platelet function test used in this study, PFA-100 with collagen and epinephrine cartridges, has been questioned with regard to response to aspirin. It is a COX-1-non-specific test, and therefore not quite suitable to detect real aspirin-resistance. High RPR by this method is not necessarily due to insufficient inhibition of COX-1, but might be caused by platelet activation through other pathways. Although high RPR by the PFA-100 method have, in some studies shown to be associated with increased risk of clinical endpoints [11], in our population, as well as in other studies, PFA-100 was not able to identify these high-risk patients [12, 33, 34]. RPR was determined only at baseline without re-testing during the follow-up period. It should also be emphasized that the method is dependent of vWF levels [13, 35]. However, in our population very few had levels below or above the reference values. vWF varies with blood type and the coagulation factors VIII and fibrinogen concentrations [21, 36, 37]. It is also uncertain whether the vWF antigen levels measured reflect vWF length.

## Conclusion

In our population of stable CAD patients on aspirin treatment, low ADAMTS13 levels were associated with high RPR and in combination with vWF associated with clinical outcome after 2 years. Beyond the properties of ADAMTS13 to cleave vWF, other mechanisms behind these associations are not clear and further investigations are needed.

## Additional file

Additional file 1: Table S1. Baseline characteristics according to clinical endpoint or no endpoint. (PDF 173 kb)

## Abbreviations

ACE: Angiotensin-converting enzyme
ADAMTS13: A Disintegrin And Metalloprotease with TromboSpondin type 1 motif, member 13
ARB: Angiotensin II receptor blockers
ASCET: Aspirin Nonresponsiveness and Clopidogrel Endpoint Trial
CABG: Coronary artery bypass graft
CAD: Coronary artery disease
CT: Closure time
CVD: Cardiovascular disease
MI: Myocardial infarction
PCI: Percutaneous coronary intervention
RPR: Residual platelet reactivity
TxB_2_: Thromboxane B_2_
vWF: Von Willebrand Factor
