# Supplementary material for: vWF/ADAMTS13 is associated with on-aspirin residual platelet reactivity and clinical outcome in patients with stable coronary artery disease
Source: Thromb J. 2017 Nov 22;15:28. doi: 10.1186/s12959-017-0151-3 (PMC5700557; doi:10.1186/s12959-017-0151-3)
Supplement: Additional file 1: Table S1. — Baseline characteristics according to clinical endpoint or no endpoint. (PDF 173 kb) [file 12959_2017_151_MOESM1_ESM.pdf]

Additional file 1 Table S1: Baseline characteristics according to clinical endpoint or no endpoint.

	Endpoint (n=73)	No endpoint (n=927)	p-value[§]
Age (years) [a]	64.2 (44.0-80.3)	62.2 (36.4-80.8)	0.074
Sex, female, n (%)	17 (23.3)	201 (21.7)	0.749
Caucasian, n (%)	72 (98.6)	896 (96.7)	0.356
Cardiovascular risk factors, n (%)			
Current smoking	20 (27.4)	183 (19.8)	0.119
Hypertension	44 (60.3)	512 (55.3)	0.409
Diabetes mellitus	22 (30.1)	178 (19.2)	**0.025**
Previous CVD[d]	57 (78.1)	578 (62.4)	**0.007**
- Previous myocardial infarction	42 (57.5)	394 (42.6)	**0.013**
Systolic blood pressure (mmHg) [b]	140±19	139±19	0.844
Diastolic blood pressure (mmHg) [b]	81±9	82±10	0.468
Body mass index (kg/m^2) [b]	27.3±4.0	27.4±3.7	0.742
Blood tests			
Total cholesterol (mmol/L) [b]	4.54±0.97	4.55±0.98	0.927
LDL cholesterol (mmol/L) [b]	2.49±0.74	2.53±0.84	0.706
HDL cholesterol (mmol/L) [b]	1.34±0.39	1.33±0.41	0.822
Triglycerides (mmol/L) [c]	1.30 (0.99, 1.89)	1.31 (0.93, 1.84)	0.897
Residual platelet reactivity, n (%)	19 (26.0)	239 (25.8)	0.967
TxB_2 (ng/mL) [a]	2.3 (0-20)	2.7 (0-21)	0.301
Medication, n (%)			
Statins	72 (98.6)	910 (98.3)	0.820
B-blockers	54 (75.0)	701 (75.9)	0.869
Calcium channel blockers	20 (27.4)	235 (25.5)	0.715
ACE-inhibitors	24 (32.9)	239 (26.0)	0.197
ARB	23 (31.5)	216 (23.5)	0.121

[a] mean (range) [b] mean±SD [c] median (25th, 75th percentiles)

[d] Previous MI, PCI, CABG, non-haemorrhagic stroke

[§] p-values refer to differences between the groups with clinical endpoint and no endpoint.

Significant p-values are highlighted with boldface

CVD = cardiovascular disease

MI = myocardial infarction

PCI = percutaneous coronary intervention

CABG = coronary artery bypass graft

TxB_2 = thromboxane B_2

ACE = angiotensin-converting enzyme

ARB = angiotensin II receptor blockers
